# The clinical burden of malaria in Nairobi: a historical review and contemporary audit

**DOI:** 10.1186/1475-2875-10-138

**Published:** 2011-05-20

**Authors:** Sandra A Mudhune, Emelda A Okiro, Abdisalan M Noor, Dejan Zurovac, Elizabeth Juma, Sam A Ochola, Robert W Snow

**Affiliations:** 1 Malaria Public Health & Epidemiology Group, Centre for Geographic Medicine Research - Coast, Kenya Medical Research Institute/Wellcome Trust Research Programme, P.O. Box 43640, 00100 GPO, Nairobi, Kenya; 2 Centre for Tropical Medicine, Nuffield Department of Clinical Medicine, University of Oxford, CCVTM, Oxford OX3 7LJ, UK; 3 Ministry of Public Health & Sanitation, Division of Malaria Control, P.O. Box 19982-00202, Nairobi, Kenya; 4 Provincial Director of Public Health & Sanitation Services, Ministry of Medical Services, P. O. Box 34349-00100, Nairobi, Kenya

## Abstract

**Background:**

Widespread urbanization over the next 20 years has the potential to drastically change the risk of malaria within Africa. The burden of the disease, its management, risk factors and appropriateness of targeted intervention across varied urban environments in Africa remain largely undefined. This paper presents a combined historical and contemporary review of the clinical burden of malaria within one of Africa's largest urban settlements, Nairobi, Kenya.

**Methods:**

A review of historical reported malaria case burdens since 1911 within Nairobi was undertaken using archived government and city council reports. Contemporary information on out-patient case burdens due to malaria were assembled from the National Health Management and Information System (HMIS). Finally, an audit of 22 randomly selected health facilities within Nairobi was undertaken covering 12 months 2009-2010. The audit included interviews with health workers, and a checklist of commodities and guidelines necessary to diagnose, treat and record malaria.

**Results:**

From the 1930's through to the mid-1960's malaria incidence declined coincidental with rapid population growth. During this period malaria notification and prevention were a priority for the city council. From 2001-2008 reporting systems for malaria were inadequate to define the extent or distribution of malaria risk within Nairobi. A more detailed facility review suggests, however that malaria remains a common diagnosis (11% of all paediatric diagnoses made) and where laboratories (n = 15) exist slide positivity rates are on average 15%. Information on the quality of diagnosis, slide reading and whether those reported as positive were imported infections was not established. The facilities and health workers included in this study were not universally prepared to treat malaria according to national guidelines or identify foci of risks due to shortages of national first-line drugs, inadequate record keeping and a view among some health workers (17%) that slide negative patients could still have malaria.

**Conclusion:**

Combined with historical evidence there is a strong suggestion that very low risks of locally acquired malaria exist today within Nairobi's city limits and this requires further investigation. To be prepared for effective prevention and case-management of malaria among a diverse, mobile population in Nairobi requires a major paradigm shift and investment in improved quality of malaria diagnosis and case management, health system strengthening and case reporting.

## Background

The intensity of malaria transmission in urban settlements of Africa is often considerably lower than the immediate peri-urban and rural surrounds [1-7] and driven largely by the presence of focal breeding sites developed for water storage or urban agriculture [8-12]. However, despite much reduced risks of acquiring malaria infections the diagnosis of clinical malaria in urban, low transmission areas remains common [13-16]. Rapid assessments of the malaria situation in four cities: Dar es Salaam, Tanzania [17], Abidjan, Cote D'Ivoire [18], Cotonou, Benin [19] and Ouagadougou, Burkina Faso [20] have highlighted the high rate of over-diagnosis of malaria among febrile patients attending clinics; the focal nature of infection risks associated with seasonal agricultural activities; and the inadequacies of routine medical statistics that should provide a realistic surveillance of malaria risks in these urban areas of sub-Saharan Africa. The authors conclude that the extent, risk factors, disease burden and appropriateness of targeted interventions in urban areas of Africa remains unknown.

Africa is expected to experience rapid rates of urbanization with 54% of Africans living in urban areas by 2030 [21]. This has the potential to radically change the landscape of malaria risk on the continent [22]. It is increasingly recognized that presumptive treatment of malaria in areas of Africa under declining transmission intensity is increasingly inappropriate [23], may increase patients risks of severe outcomes from un-diagnosed conditions [24] and increase unnecessary expense on limited public sector drug budgets or patients who have to pay for medicines. A multi-sectoral technical consultation on urban malaria in 2004 led to the Pretoria Statement, that stated the need to improve our understanding of the malaria risk extent, burden, diagnosis and targeted drug delivery in urban settings [6]. Unfortunately, the reliability and completeness of routine health statistics in many African urban settings remains poor [13].

The city of Nairobi, Kenya has an interesting malaria history and the extent of locally acquired transmission has not been formally defined for over 50 years. Nairobi is located 1,795 m above sea level with a temperate climate and low temperatures (that can drop to 10°C in June/July), representing sub-optimal conditions for sporogony in the Anopheles [25]. The cold seasonal temperature in Nairobi is thought to limit the development of the *Plasmodium falciparum *sporozoite stage in the salivary glands of the mosquito vector; however windows of transmission potential do exist within an average year and vary between years. The temperature limiting effects of transmission in Nairobi have largely underpinned the recent controversy on the likelihood of transmission of malaria within Nairobi and whether clinical malaria can be acquired within the city limits [26-30]. In an effort to improve the understanding of the malaria burden in Nairobi, this paper combines historical evidence of the reported clinical burden of malaria from the early 1900's as it grew in size to the present day and a contemporary audit of diagnoses made at government supported clinics across the city in 2010 to augment inadequate information systems reported data.

## Methods

### Review of historical malaria case reports and recent health information system data

Reports of malaria cases since 1911 were reviewed from annual medical reports, authored by the Director of Medical Services for the Colony and Protectorate of Nairobi (1908-1964; missing 1909, 1911-1915) and the Medical Officer of Health, for the Nairobi Municipality (1930-1964; missing 1940-1945) retrieved from the Kenya National Archives Library, the Ministry of Health Library and the Macmillan Memorial Library in Nairobi. These extracted data were assembled to define reported malaria cases and triangulated with other published sources that described the various epidemics. Contemporary information on out-patient case burdens due to malaria were assembled from annual reports from the Ministry of Health's National Health Management and Information System (HMIS). Data are compiled by the health service information subsystem and are routinely collected through a network of facility units distributed throughout the country [31-34].

### Health facility audit 2010

**Figure 1:**
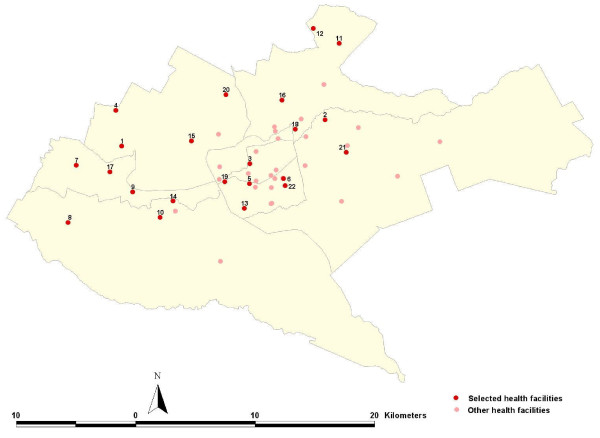
**A division map of Nairobi showing the location of public health facilities in Nairobi**. The red dot represents health facilities selected for the survey and the pink dots represent other health facilities run by MoMS or the LA in Nairobi. Surveyed facilities are as follows: 1 = Kangemi Health Centre; 2 = Dandora 2 Health Centre; 3 = Pumwani Dispensary; 4 = Lower Kabete; Health Centre; 5 = Kaloleni Sub-Health Centre; 6 = Jericho Health Centre; 7 = Waithaka Health Centre; 8 = Karen Health Centre; 9 = Ngong Road Health Centre; 10 = Langata Health Centre; 11 = Kahawa Health Centre; 12 = Kamiti Health Centre; 13 = Railways Training School Clinic; 14 = Kibera DO Health Centre; 15 = Westlands Health Centre; 16 = Karura Health Centre; 17 = Riruta Health Centre; 18 = Kariobangi Health Centre; 19 = Locomotive Health Centre; 20 = Mji Wa Huruma Dispensary; 21 = Soweto/Kayole Dispensary; 22 = Makadara Health Centre.

A database of geo-located health facilities in Kenya [35] was used to identify all out-patient service providers in Nairobi that are supported by the Ministry for Medical Services (MoMS) or managed by the Local Authority (LA) and provide general out-patient care, i.e. are not specialist centres for TB, mental health or reproductive health or attached to district or tertiary referral hospitals. Of the universe of 52 MoMS or LA primary-level out-patient providers, 22 were randomly selected to form the basis of the present study (Figure 1). Survey tools were adapted from those used in Kenya to define the quality of malaria case-management [36,37] and included: a) facility audit of available drugs, diagnostics, malaria guidelines and job aides, record keeping materials, weighing scales and other supporting commodities; b) interview schedule with health workers performing case-management on the day of the survey to determine health workers' demographics, exposure to training in malaria diagnosis and treatment and knowledge of standard recommended treatment protocols; and c) review of available attendance and laboratory records kept at the facility over the preceding 12 months to tally the monthly numbers of diagnoses made that included "malaria", those that did not include a diagnosis of "malaria", the number of diagnostic tests performed and the number reported positive among attendees. Five data collectors were recruited and trained in all survey procedures and the survey was undertaken between the 16^th ^August and 6^th ^September 2010. Data were entered from questionnaires and checked in Excel (Microsoft, USA) and analysis performed in STATA, version 11 (Stata Corp, College Station, Texas).

### Ethical approval

Ethical approval was provided by the Kenyatta National Hospital/University of Nairobi Ethics and Research Committee (reference number KNH-ERC/A/383).

## Results

### The historical evidence of clinical malaria in Nairobi 1911-1960's

Malaria has been a reported clinical problem since Nairobi was first established as a colonial headquarters in 1905. Over 14,000 malaria cases were recorded in Nairobi in 1913 [38]. Malaria cases treated in government hospitals fluctuated between 2,500 and 3,600 per year between 1917 and 1919. The malaria burden remained high between 1921 and 1925 in Nairobi, with one major outbreak in 1922. For non-epidemic years 1916-1925 an average of 15.5 malaria deaths were recorded annually in government clinics across Nairobi accounting for approximately 4.1% of all hospital reported deaths across the country [38]. In 1926 [38,39], 1935 and 1940 [39,40] Nairobi suffered from malaria epidemics of substantial proportions. In 1926, more than 12,000 cases were treated in hospitals and health centres around Nairobi and the Medical Officer of Health reported 130 deaths due to malaria [38]. The epidemic in 1926 renewed the efforts to control malaria in Nairobi and its surveillance was improved [38]. The political concern generated by this epidemic resulted in a decision by the government to make malaria a notifiable disease in 1930 [39,41]. All medical practitioners, including those in private practices, were required by law to report all cases treated for malaria to the Medical Officer of Health. Laboratory confirmation was required for any cases deemed to be due to infections acquired locally. Malaria infections suspected to have been acquired in Nairobi were classified as 'locally acquired infections,' 'Nairobi infections,' or as 'contracted in Nairobi among residents' [41].

**Figure 2:**
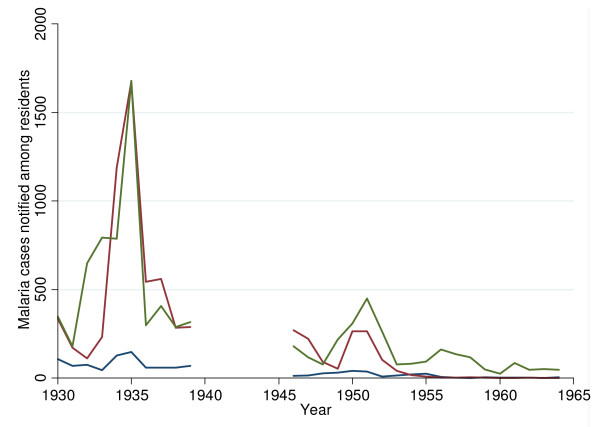
**Malaria morbidity among residents in Nairobi between 1930 and 1964**. The blue line represents malaria cases among Europeans; the red line represents cases among Asians and the green line that among Africans. (Data assembled Annual Reports of the Medical Officer of Health 1930-1964; missing 1940 - 1945).

The malaria epidemic of 1935 followed a high incidence of malaria in 1934 (Figure 2). By the end of March 1935 there was a sudden rise in the number of cases and the epidemic reached its peak in May and then experienced a steep decline and was over by August. A total of 3,500 malaria cases were recorded, 96% of which were among Asian or African residents who made up all of the 58 deaths recorded during this period [41]. By 1939, there were fewer cases of malaria reported and the death rate from malaria was the lowest recorded up to this point at 0.24 per 1000 persons [39]. The malaria epidemic in 1940 caused a total of 8,324 malaria cases (6,342 cases among Nairobi residents) and 62 malaria deaths [39]. The Army stationed in Nairobi was largely spared during this epidemic as strict measures had been undertaken to enforce regular compliance to the use of preventive measures and prophylactic drugs [42]. The European population appeared to be largely unaffected by the 1940 epidemic.

Between 1930 and 1964, annual reports indicate that the period with the highest number of malaria cases occurred between March and May. This seasonal pattern of malaria was consistent throughout the reports examined. The number of malaria cases would begin to rise in February and March and the peak would occur between the months of April and July. Overall, the trend since 1911 has been of a declining incidence punctuated with epidemics. Between 1930 and 1939 malaria accounted for 3.7% of the total number of deaths in Nairobi while from 1946 to 1964 it accounted for 1.2%. Notified malaria cases showed a significant decrease of autochthonous malaria from an annual average of 1,182 cases in the 1930s, to 317 cases in the 1940s to 250 in the 1950s and finally 49 cases in the 1960s during a period when the numbers of Nairobi residents had increased 35 times since the 1930's [41]. Various reports suggested that aggressive control using recurrent operations such as oiling, cleaning of streams and drains, grass cutting and spraying with dichlorodiphenyltrichloroethane (DDT) may have accounted to a large extent for this decline. Malaria was perceived to be a significant public health threat and between 1950 and 1961 the Municipal Council continued to invest on average 3.9% of the total health expenditure during this period on direct vector control [41,42].

### HMIS reports of malaria 1970-2008

The accuracy and completeness of health information reporting on malaria across Nairobi began to decline from the 1970's and routine HMIS data are hard to locate for the city's case burdens. While it was accepted during the 1970's that transmission was possible in low lying areas of Nairobi [26] the extent of over-diagnosis of locally acquired infections became a topic of investigation. A study conducted between October 1969 and June 1970 at the Kenyatta National Hospital (KNH) among 85 patients with suspected or 'clinical' malaria found only one patient with parasitologically confirmed malaria [26]. In 1980, only 5% of adults admitted to the KNH with a diagnosis of malaria were finally confirmed as *P. falciparum *cases through microscopy [43].

A study of malaria out-patient diagnoses reported to the central Health Information System by public health facilities nationally showed that only 35% of expected reports between January 1996 and December 2002 were reported [44]. The Annual Health Sector Report for 2008 on the reporting rates for outpatient morbidity found Nairobi the worst with 37% for timeliness and 42% for completeness [34]. In 2007 only 3 health facilities reported any out-patient morbidity statistics. Despite the incomplete nature of the information, malaria remains the second most commonly diagnosed disease among attendees to out-patient clinics after respiratory tract infections between 2001 and 2008. Malaria was reported as responsible for out-patient consultations in 10.6% of attendances in 2001 (17,567 reported cases), 16% in 2005 (90,214 reported cases), 18% in 2006 (126,604 reported cases) and 16% in 2008 (120,782 cases) [31,32,45].

### Health facility audit 2010

Of the 22 health facilities surveyed four (18%) were run by the MoMS and 18 (82%) were run by the LA. Twenty facilities had a functioning thermometer on the day of the survey. At least one type of weighing scales was available at each facility on the day of the survey although these varied in application for different weight groups (Table 1). Current malaria policy recommends that all facilities have parasitological diagnosis either by microscopy or Rapid Diagnostic Tests (RDTs) [45]. Fifteen facilities (68%) routinely provided microscopy; none of the facilities had RDTs; on the day of the survey only 14 facilities (64%) had functioning microscopy services available. Eight facilities had at least one laboratory staff who had received in-service training in malaria microscopy, while six had at least one laboratory staff member who had been trained in RDT use.

**Table 1:** Characteristics of the 22 health facilities in Nairobi (n, (%))

Equipment and services at health facility	
Weighing scale (any)	21 (95.5)
Infant hanging scale	17 (77.3)
Hanging Salter scale	7 (31.8)
Adult scale	14 (63.6)
Bathroom scale	15 (68.2)

Thermometer	20 (90.9)

Separate laboratory	15 (68.2)

Functional microscopy	14 (66.7)

**Record Keeping**	

Stock/Bin cards for AL	14 (63.6)

AL dispensers book	16 (72.7)

Monthly summary for malaria medicines	16 (72.7)

Out-patient record books	12 (54.5)

Laboratory record books	12 (54.5)

**Wall charts & Guidelines**	

Malaria Guideline (Version 2)	15 (68.2)

Malaria Management chart booklet	8 (36.4)

Algorithm for <5 years fevers	7 (31.8)

AL dispensing and dosing schedule	12 (54.6)

Malaria OPD algorithm for older children & adults	4 (18.2)

Integrated management of childhood illnesses chart	11 (50)

**Availability of AL on the survey day**	

Any tablets of AL	19 (86.4)

AL 6 tablets pack	16 (72.7)

AL 12 tablets pack	18 (81.8)

AL 18 tablets pack	16 (72.7)

AL 24 tablets pack	16 (72.7)

Stock out in last three months*	

Any tablets of AL	19 (86.4)

AL 6 tablets pack	10 (55.6)

AL 12 tablets pack	9 (50)

AL 18 tablets pack	8 (44.4)

AL 24 tablets pack	7 (38.9)

**Availability of other antimalarial drugs on the survey day**	

Amodiaquine (any formulation)	1 (4.5)

Sulfadoxine-pyrimethamine (any formulation)	14 (63.6)

Quinine (tablets)	12 (54.5)

Quinine (injection)	9 (40.9)

Artemether injection	0 (0)

*data unavailable from 4 facilities

Fourteen facilities (64%) had at least one of the three widely disseminated anti-malarial drug record books (Table 1). Of those with drug records, the level of completeness was high with 12 (86%) complete stock bin cards, 15 (88%) complete dispenser's book and 11 (65%) complete monthly summary forms. Fifteen facilities 68%) had out-patient record books available on the day of the survey, however only ten (46%) health facilities had contiguous data for the 12 months; nine (41%) facilities had incomplete out-patient records available. Six (27%) facilities did not have the original monthly reports at the facility and their data were retrieved from district headquarters, which keeps a copy of their data. Of the 15 facilities that had a functioning laboratory, only twelve had complete laboratory registers available for review for the preceding 12 months.

Health facilities were also assessed for availability of guidelines and wall charts that act as job aids for malaria case management. Most (68%) facilities had the malaria guidelines prepared for health workers in 2008 [46] and the availability of the other guidelines was incomplete (Table 1). There was also a low coverage of wall charts with less than half of the facilities having any of the three wall charts that were in circulation nationally (Table 1). Nineteen health facilities had AL in stock on the survey day. Most sites had the different pack sizes available with 16 (73%) having the 6, 18 and 24 tablet packs in stock and 18 (82%) facilities had the 12 tablet packs in stock (Table 1). Thirteen (59%) facilities had a documented stock out of at least seven consecutive days of any of the AL packs in the past three months, May 2010 to July 2010 (Table 1).

On the day of the survey 29 out of the 31 health workers who were attending to out-patients were interviewed. Two health workers declined to participate. The health workers interviewed were either nurses (23%) or clinical officers (77%). Among the health workers, 20 (69%) were trained in malaria case management including the use of AL. The malaria case management training was received by one health worker in 2006; two in 2008; five were trained in 2009; and 12 in 2010. The only health worker with RDT training received the training in 2009. Eighteen (62%) health workers had access to the malaria guideline; seven (24%) had access to the malaria management chart booklet; and 11 (38%) had access to the Integrated Management for childhood Illnesses (IMCI) guideline. Supervision on malaria case management was low with only nine (43%) of the health workers having had a supervisory visit in the last three months. Of those who had a supervisory visit malaria case management was the topic for nine health workers. Topics covered in the malaria case management supervision included a review of malaria records, discussion with supervisor on case management, and observed consultations in six cases (67%), feedback was provided following 67% of supervisions.

Health workers were asked to provide a response to a series of questions related to recommended treatment regimens for malaria and the responses showed some variation between health workers in their knowledge of first line and second line recommended therapies for uncomplicated malaria (Table 2). Each of the 29 health workers was presented with a series of statements to determine their perceptions and acceptance of malaria parasite testing and treatment. Malaria parasitological testing was thought a necessary requirement of febrile patients in Nairobi by 48% of health workers and a minority (17%) reported they would still treat for malaria even when presented with a negative diagnostic result (Table 2).

**Table 2:** Responses to questions on recommended treatment and reported use of diagnostics by 29 health workers based in 21 health facilities in Nairobi

1^st ^line treatment for children below 5 kg with uncomplicated malaria	N = 29
AL	11 (37.9%)

Quinine	15 (51.7%)

Amodiaquine	2 (6.9%)

Don't know	1 (3.4%)

**1^st ^line treatment for children > 5 kg and adults with uncomplicated malaria**	

AL	27 (93.1%)

SP	2(6.9%)

**2^nd ^line treatment for children below 5 kg and adults with uncomplicated malaria**	

AL	4 (13.8%)

Quinine	23 (79.3%)

DuoCotexin	1 (3.4%)

Don't know	2 (6.9%)

**2^nd ^line treatment for children below 5 kg and adults with uncomplicated malaria**	

AL	3 (10.3%)

SP	1 (3.4%)

Quinine	(44.8%)

DuoCotexin	2 (6.9%)

DHA-PPQ (dihydroartemisinin-piperoquine	8 (27.6%)

Lapdap (chloroproguanil-dapsone)	1 (3.4%)

Don't know	1 (3.4%)

**Responses to questions on diagnostics - proportion agreeing with statements**	

All febrile patients in Nairobi should be tested for malaria	14 (48.3%)

Only febrile patients who recently travelled outside of Nairobi should be tested for malaria	13 (44.8%)

Most febrile patients in Nairobi with negative RDT should be still treated for malaria	4 (13.8%)

Most febrile patients in Nairobi with negative Blood Slide should be still treated for malaria	5 (17.2%)

### Assembling the malaria case burdens at 15 clinics August 2009-July 2010

Monthly tallies of all diagnoses were possible for all facilities; however, actual records of diagnosis were not kept at one facility (Makadara Health Centre). Retrieving the out-patient records proved time-consuming at each facility as non-current record books were scattered around the facility and for six facilities data had to be retrieved from district-level headquarters as these had not been returned to the facility. Among the 20 facilities where diagnosis-specific records were retrievable, out-patient consultations were tallied for 201 (83.8%) of a possible 240 facility-months. At five facilities it was possible to assemble records for all 12 months, however at one facility only six months of records could be assembled (Table 3). Across the recorded months a total of 380,335 out-patient consultations were documented of which 37,352 (9.8%) were classified as including a presumptive malaria diagnosis. Approximately equivalent proportions of out-patients aged less than five years (8.7%) or above five years (10.6%) were diagnosed with malaria (Table 3). The highest proportion of malaria diagnoses made amongst all age groups attending clinic between 2009-2010 was documented at the Karura Health Centre (31.6%; Table 3; Figure 1 code 16) and the lowest documented proportion was recorded at Kibera DO Health centre (1%; Table 3; Figure 1 code 14). Out of the 16 facilities where laboratory services were available, it was possible to assemble records for all 12 months at 10 facilities. Three facilities were missing one month of data while in the second and third facilities three and four months of data were missing respectively. Of the 37,544 blood samples sent to the 16 facilities with a laboratory, 5,540 (14.6%) malaria blood films were recorded as positive (Table 3). This includes both age groups of patients and the value varied from as low as 1.6% in Westlands (Table 3; Figure 1 code 15) to 31.4% in Kamiti (Table 3; Figure 1 code 12).

**Table 3:** Record reviews August 2009-July 2010 for 21 facilities (Madakara code 22 not shown as no data available) where a diagnosis was provided in out-patient record books

Facility Map code (Figure 1)	Total Months reviewed (Missing months)	Total under 5 OPD burden	Total presumed malaria diagnoses <5 years (% of all OPD)	Total over 5 OPD Burden	Total Presumed Malaria diagnoses >= 5 years (% of all OPD)	Total all ages slides performed	Total all ages Slides reported positive (%)
Kangemi 1	12	8869	265 (3.0)	16117	639 (4.0)	1626	120 (7.4)

Dandora 2	12	10004	556 (5.6)	14548	769 (5.3)	1131	92 (8.1)

Pumwani 3	11 (June)	7139	1157 (16.2)	17101	2896 (16.9)	1209	204 (16.9)

Lower Kabete 4	12	5432	654 (12.0)	7051	997 (14.1)	No Lab	No Lab

Kaloleni 5	9 (May - July)	5098	408 (8.0)	6804	1353 (19.9)	No Lab	No Lab

Jericho 6	10(Aug, Dec)	33328	2140 (6.4)	16630	1942 (11.7)	4312	195 (4.5)

Waithaka 7	8 (Aug - Oct; Apr)	9985	427 (4.3)	12141	1248(10.3)	2036	93 (4.6)

Karen 8	6 (Aug - Oct; Jan - Mar)	4477	361 (8.1)	9441	292 (3.1)	1404	359 (25.6)

Ngong' 9	9 (Aug; Jan; Feb)	7510	-	11603	964 (8.3)	No Lab	No Lab

Langata 10	9 (Aug - Oct)	8127	621 (7.6)	12138	1163 (9.6)	3547	825 (23.3)

Kahawa 11	11 (Mar)	12861	1058 (8.2)	18631	1556 (8.4)	2488	105 (4.2)

Kamiti 12	9 (Aug, Feb, July)	7475	679 (9.1)	13234	1393 (10.5)	7041	2213 (31.4)

Railway 13	11 (June)	3514	713 (20.3)	4698	1348 (28.7)	No Lab	No Lab

Kibera 14	12	4947	36 (0.7)	4791	59 (1.2)	1473	227 (15.4)

Westlands 15	7 (Oct, Dec, May- July)	4823	875 (18.1)	9974	1557 (15.6)	807	13 (1.6%)

Karura 16	12	3426	1233 (36)	5907	1600 (27.1)	No lab	No lab

Riruta 17	-	-	-	-	-	3404	288 (8.5%)

Kariobangi 18	11 (Mar)	7713	1152 (14.9)	17241	1865 (10.8)	4592	601 (13.1%)

Locomotive 19	8 (Aug - Nov)	2635	437 (16.6)	11216	1505 (13.4)	1744	129 (7.4%)

Huruma 20	11 (Feb)	9262	364 (3.9)	7161	291 (4.1)	No lab	No lab

Soweto/Kayole 21	11 (Nov)	2492	684 (27.4)	-	867 (-)	730	76 (10.4)

**Total**	**201**	**159,117**	**13,856 (8.7)**	**221,218**	**23,496 (10.6)**	**37,544**	**5,540 (14.6)**

## Discussion

Despite unfavourable climatic conditions, Nairobi was historically an area where malaria was a common clinical problem with recurrent epidemics through to the end of the 1940s (Figure 2). Nairobi's population grew from an estimated 17,000 in 1910 [47] to approximately 300,000 by 1964 [48]. This growth brought with it changes in settlement patterns, regulations governing where people could live [49] and a growth in urban infrastructure. By the 1960s, the recorded incidence of malaria had declined significantly (Figure 2) suggesting a receptive location for transmission, but effectively controlled. The documented efforts by the city council during this period are impressive and malaria was a priority for the administration of the time, ensuring that cases were confirmed and formed part of a notifiable disease surveillance system [41]. Precisely how complete disease reporting was remains impossible to judge.

Between 1961 and 1999 Nairobi's population expanded to an estimated 2.14 million residents, living in a slightly expanded area to the boundaries of Nairobi of the 1960's. Population growth was driven by large-scale national in-migration for work and sub-regional migrants seeking refuge from war-torn neighbouring countries. This population pressure led to the early expansion of urban slums [49,50]. Throughout this period very little information is available on the clinical and biological threats posed by malaria across the city. The coverage and completeness of health statistics declined and no documented evidence was available to suggest any city council or Ministry of Health allocations to malaria prevention or vector control specific to the city limits. This period probably reflects a more international decline in vertical programme interest in malaria following the abandonment of the Global Malaria Eradication Programme in Africa and the subsequent inclusion of malaria as part of general care provision through initiatives such as Primary Health Care and IMCI.

Health Information systems continued to provide challenges for the investigation of the extent and burden of malaria through to 2008. In 2007, only three health facilities reported to the Government's national HMIS. Despite the vagaries and incompleteness of the HMIS data what emerges is that malaria remains to be a very common diagnosis made at out-patient facilities across the city, second only to respiratory tract infections and contributing between 9-16% of the annual out-patient burden. To investigate the facility-level morbidity reports in more detail, records at 22 facilities were reviewed for a period of one year August 2009-July 2010. Following repeated searches and follow-up at district headquarters complete record books for 12 months were only available for 10 facilities reflecting a more basic weakness in the HMIS system beyond simply reporting to headquarters. At 21 health facilities a total of 201 months of out-patient information was available for review (Table 3). Of 159,117 paediatric diagnoses made, 13,856 (8.7%) were recorded as malaria; among adults 10.6% of 22,496 diagnoses were recorded as malaria. These reported burdens are not dissimilar to those noted from incomplete HMIS data between 2001 and 2008 and support a general view of patients [51] and health workers [52] resident and working in Nairobi that malaria is one of the most common morbid burdens they face. As reported from a wide variety of urban settings across Africa [17-20] and in Nairobi [26,43,52], the incidence of presumed versus confirmed malaria are often very different. At 14 facilities with a functioning laboratory and available record books of microscopy the results of 37,544 slides taken during the period of review showed that 14.6% were recorded as positive, ranging from 4% to 31% between facilities with half of the surveyed facilities reporting slide positivity rates above 10%. These data are hard to interpret without any sense of criteria used at each facility to request parasitological diagnosis or a measure of the accuracy of slide reading and recording. Nevertheless there is a strong suggestion that parasitologically-confirmed clinical malaria does present to health facilities in Nairobi. No documentation is ever made on travel histories, a significant risk factor for the diagnosis of malaria in Nairobi [52] and it was impossible to link records in out-patient registers to records in laboratories where these existed. This lack of detail continues to hinder reliable estimations of malaria risks in Nairobi and differs significantly in the reported efforts made during the 1930's to ensure that autochthonous versus imported slide confirmed malaria were documented for each patient [41].

Accuracy of diagnosis is key to appropriate management of fevers in low transmission settings. Efforts are underway to scale up and improve national government run health facilities' abilities to reliably diagnose malaria. However, its success will depend on adherence to test results, adequate supplies of rapid diagnostic tests where laboratories do not exist and supplies to support microscopy where this is available. Both requirements demand appropriate training and supervision and a reliable information and commodity supply chain. The findings from 22 clinics and 29 health workers indicate weaknesses in drug supply (Table 1), information communication (many records not available at the facility or a means to record data, Table 1) and adequacy of previous in-service training on nationally recommended therapies and diagnosis. If clinical malaria is to be treated as a notifiable disease to target resources and identify foci for investigation and vector control, a significant investment is required to improve the existing health system deficiencies.

Only 52 government or local authority run clinics providing malaria treatment services to Nairobi's currently estimated population of 3.14 million people [53]. As suggested from various household surveys the majority of Nairobi residents seek treatment for malaria outside the formal health care sector from private practitioners, mission and NGO run clinics and the retail sector [27,54,55]. Even with a functioning formal sector disease reporting and diagnostic service the use of treatment sectors outside of the formal government or LA managed sector would provide major limitations to the completeness and accuracy of a malaria notification system.

## Conclusions

The possibility of locally acquired malaria infections in Nairobi remains a moot issue. *Anopheles arabiensis *larvae have been identified extensively across the city [Noboru Mikanawa, personal communication; 28, 56]. Studies of human infection prevalence among communities in the early 1980's [29] and school children in 2009 [57] from localities proximal to the health facilities shown in Figure 1 demonstrated *P. falciparum *infection prevalence between 2 and 14%. The specific identification of locally acquired versus imported infections has not been established and no study has identified sporozoite positive adult vectors in the city since the 1920s [38]. As such the risks posed by local malaria transmission remain uncertain but from combined circumstantial evidence, including the reported slide positivity at 14 clinics between 2009-2010, clearly plausible. However, should transmission exist, responses to its prevention should be adapted to suit the epidemiological patterns that prevail. The promotion of universal coverage with insecticide-treated nets would be an inappropriate use of resources. A more intelligent approach to identifying, controlling and monitoring foci of transmission is probably a far better recommendation. The only entry point for this more reasoned approach to malaria prevention in Nairobi demands a rigorous case-detection system. This will require a major investment in the efficiency of HMIS, in-service training to effect a paradigm shift in how malaria is diagnosed, documented and reported across all sectors of health service provision and a response/investigation mechanism to meet the implicit needs of those who do report possible foci of transmission. While Kenya has made some progress in tackling the high disease burden across some stable endemic malaria conditions of rural communities [58] though a national strategy developed to meet the needs of these communities it will require novel innovation to tackle existing high population density, very low transmission settings such as the city of Nairobi over the next 20 years.

## Competing interests

DZ and RWS have received honoraria from Novartis Pharma for presenting and chairing, respectively, at their national malaria control programme best practice workshops in Africa. All other authors declare no competing interests.

## Authors' contributions

SAM and RWS designed the study. SAM undertook the data collection and record reviews and wrote the first draft of the manuscript. EAO, AMN and DZ provide scientific guidance at the design stage and in the analysis of the primary data. EJ and SAO provided help and stewardship in collecting the facility level data. All authors contributed to the writing and completion of the final manuscript.
